# Epidermolysis bullosa in a mother-infant dyad

**DOI:** 10.1093/omcr/omad124

**Published:** 2023-11-28

**Authors:** R R Prashanth, Pramod Dhanraj Kamble, Abhilasha Kumari, Anitha Haribalakrishna, Sunanda Arun Mahajan

**Affiliations:** Department of Neonatology, Seth G.S. Medical College and King Edward Memorial Hospital, Maharashtra, Mumbai, India; Department of Dermatology, Seth G.S. Medical College and King Edward Memorial Hospital, Maharashtra, Mumbai, India; Department of Neonatology, Seth G.S. Medical College and King Edward Memorial Hospital, Maharashtra, Mumbai, India; Department of Neonatology, Seth G.S. Medical College and King Edward Memorial Hospital, Maharashtra, Mumbai, India; Department of Dermatology, Seth G.S. Medical College and King Edward Memorial Hospital, Maharashtra, Mumbai, India

## Abstract

Epidermolysis Bullosa is an inherited mechanobullous disorder which presents in the neonatal period as blistering skin lesions. In this case report, we describe an uncommon presentation of Epidermolysis Bullosa Simplex in a term infant, weighing 2640 g, born to a mother who was also diagnosed with Epidermolysis Bullosa Pruriginosa during the course of the evaluation of her newborn. The clinical situation presented us with a unique dilemma with regard to routine newborn care practices including handling, skin and diaper care. Though the presentation was typically characteristic of EB, we illustrate with images the diagnostic modalities and challenges faced in the hospital while caring for this fragile skin in a low and middle-income country’s neonatal intensive care unit. This is the first reported case of a neonate with Epidermolysis Bullosa Simplex born to a mother with Epidermolysis Bullosa Pruriginosa.

## INTRODUCTION

Epidermolysis Bullosa (EB) is an inherited mechanobullous disorder with an incidence of 1: 50 000 live birth/year [1]. In this case report, we describe an uncommon presentation of epidermolysis bullosa simplex (EBS) in neonates born to a mother with Epidermolysis bullosa pruriginosa (EBP). We illustrate the diagnostic modalities and challenges in the treatment of fragile skin neonate in a low and middle-income country.

## CASE REPORT

**Figure 1:**
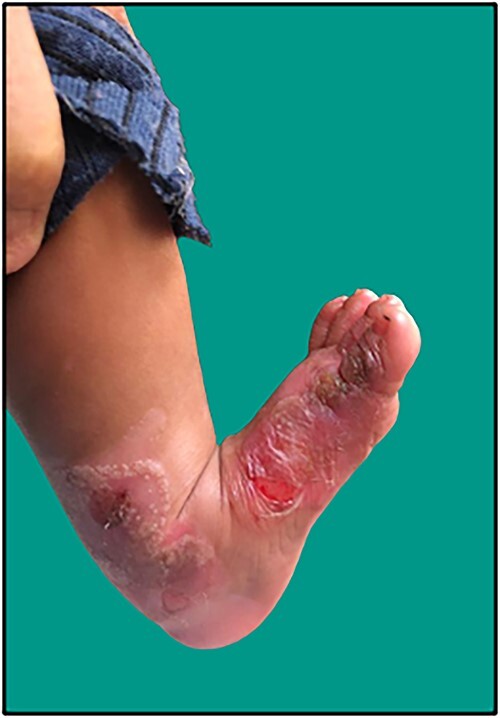
Large, ruptured bulla with clean erosion over the medial aspect of bilateral feet and medial malleolar region in the neonate.

24-year-old primigravida vaginally delivered a male neonate of 37 weeks gestation with a birth weight of 2640 grams and required no resuscitation. Rest anthropometry was also normal. Examination after birth revealed a small blister over the ring finger of the left hand followed by blisters over both shins of medial malleoli, dorsal aspect of both feet, all knuckles, flexural areas of both wrists, neck and around perioral regions which developed over the next 2 days (Fig. 1). These blisters localized to sites of mechanical trauma and were well defined with few of them spontaneously eroding. There was neither any systemic involvement nor of any mucous membrane, scalp, nails, oral cavity, palms and soles.

**Figure 2:**
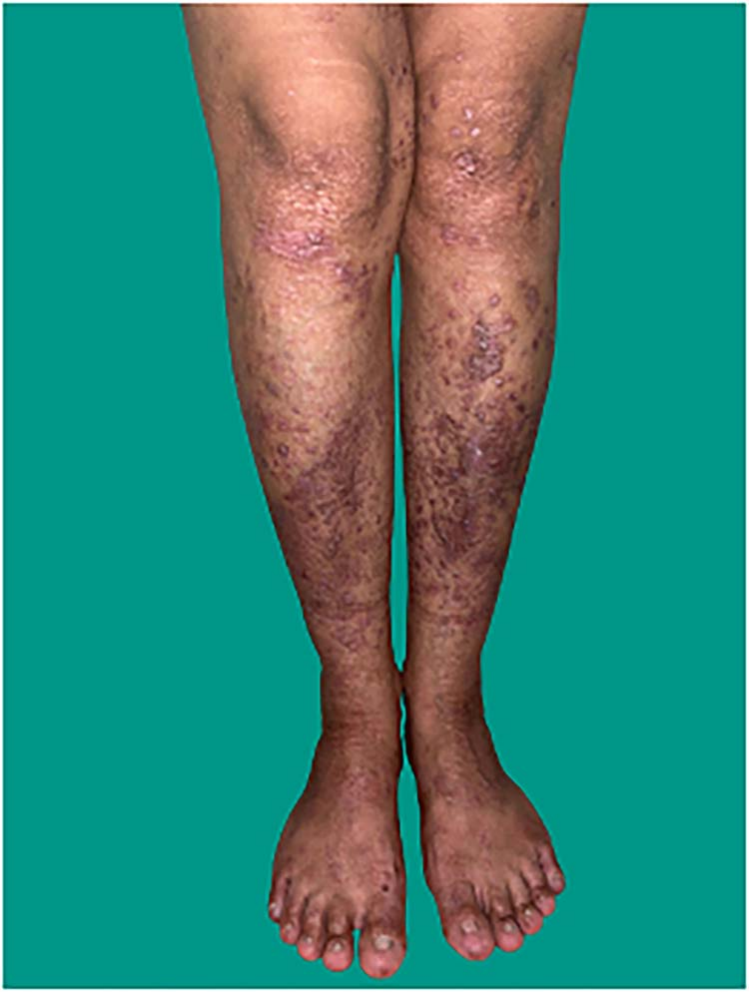
Multiple pruritic lichenified papules and plaques with milia over knees and shins (mainly over trauma-prone sites) and albopapuloid papules over the lumbosacral region with anonychia of finger and toes in the mother.

The mother had similar blistering lesions of lesser severity followed by hyperpigmented. Erythematous scarring associated with itching over trauma-prone sites from her childhood and nail deformities in her hands and feet that were undiagnosed (Fig. 2). There were no other family members with similar illnesses. The distribution of lesions and absence of mucosal involvement ruled out other differentials like bullous impetigo and neonatal pemphigus. Systemic associations were ruled out with ultrasonography of the abdomen, echocardiography and neurosonogram.

**Figure 3:**
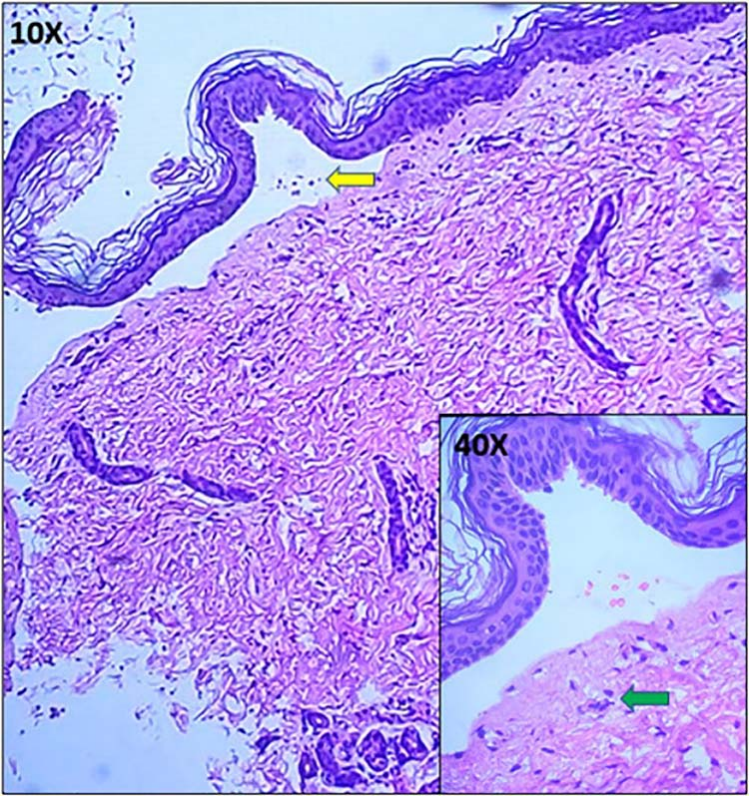
Hematoxylin and Eosin image of the neonate showing subepidermal split (arrow) with pauci immune infiltrate (arrow) in the upper dermis (Inset).

**Figure 4:**
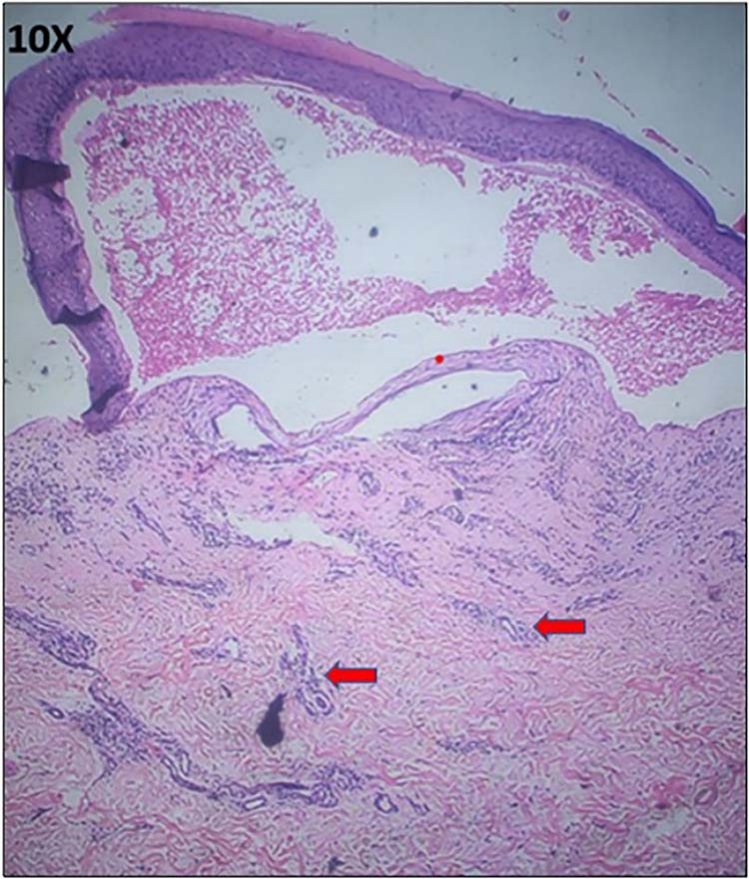
Hematoxylin and Eosin image of the mother’s biopsy from vesicle showing subepidermal split with thickened dermal vessels and perivascular lymphohistiocytic infiltrate (arrow).

**Figure 5:**
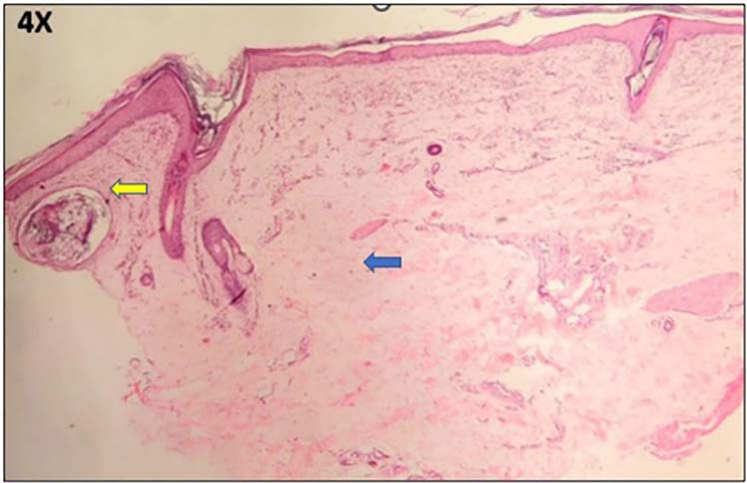
Hematoxylin and Eosin image of the mother’s biopsy from the hypertrophic plaque showing dermal fibrosis (left arrow) and milia (middle arrow).

**Figure 6:**
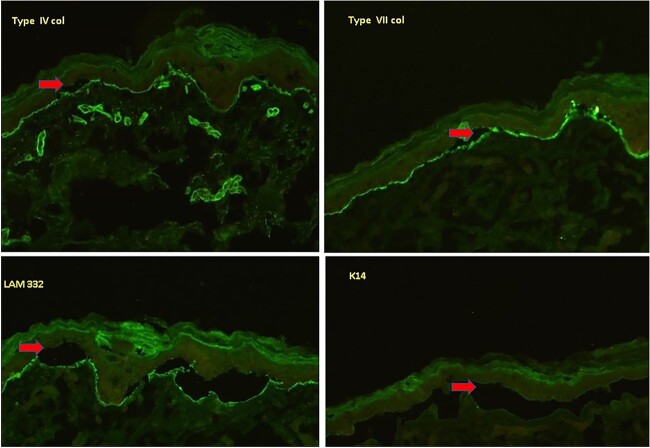
Immunofluorescence image of the neonate showing the subepidermal split (arrow) with staining of Type VII, Type IV, and Laminin 332 seen on the dermal side of the split suggestive of EBS.

**Figure 7:**
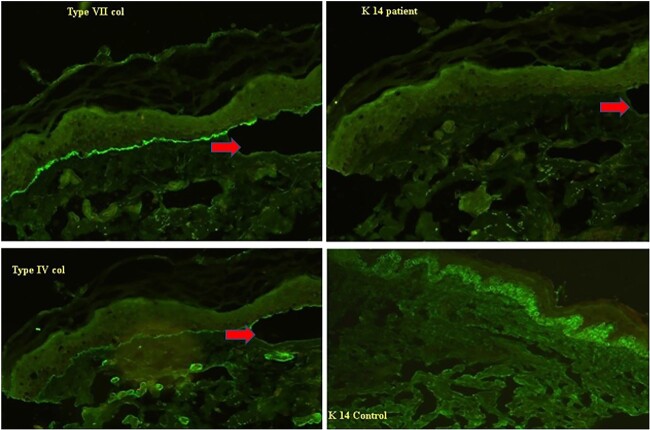
Immunofluorescence image of the mother showing the subepidermal split (arrow) with staining of Type VII, Type IV, and Laminin 332 seen on the epidermal side of the split and reduced intensity of K14 staining as compared to normal skin suggestive of a milder variant of Dystrophic EB.

Skin biopsy was performed on the mother-neonate dyad and sent for Hematoxylin and eosin (H&E) stain, immunofluorescence (IF) and antigen mapping. The H/E report of the neonate showed a subepidermal split with pauciimmune infiltrate (Fig. 3). IF and antigen mapping showed subepidermal split with staining of Type VII, Type IV and Laminin 332 on the dermal side suggestive of EBS (Fig. 4). The H/E report of the mother showed subepidermal infiltrate with perivascular lymphohistiocytic infiltrate and fibrosis in the dermis (Figs 5, 6). IF and antigen mapping showed similar results on the epidermal side and reduced intensity of K14 staining suggestive of a milder variant of Dystrophic Epidermolysis bullosa (DEB) (Fig. 7).

**Figure 8:**
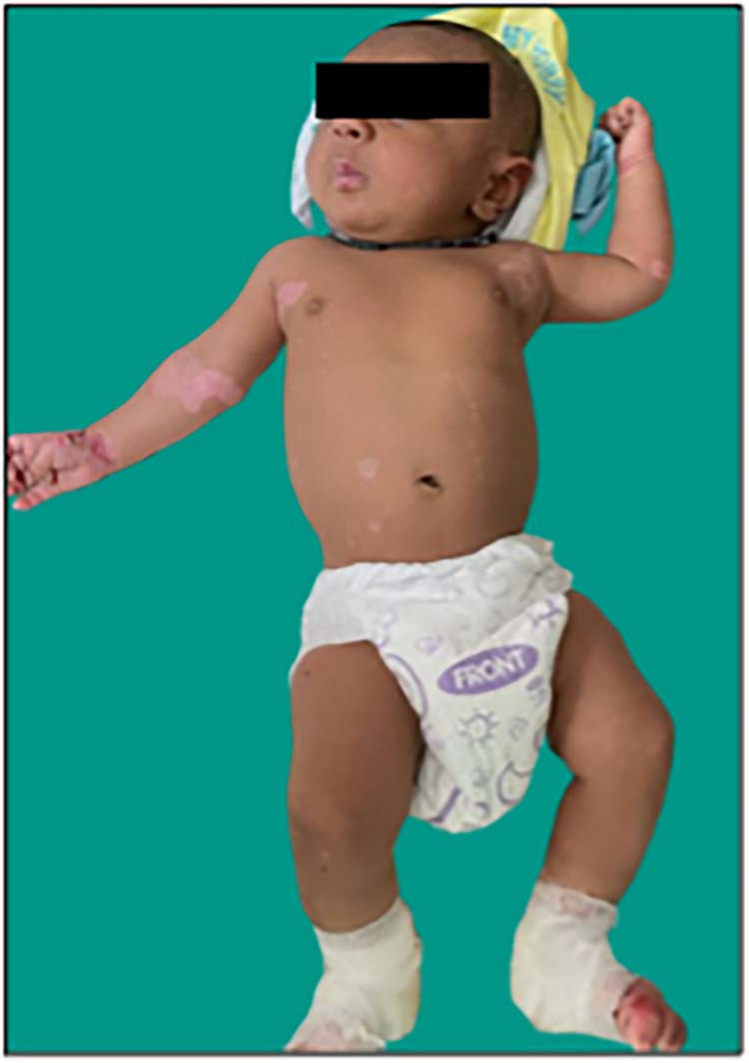
Triple layered dressing over bilateral feet of the neonate.

We used triple-layer dressing. (Fig. 8). The first layer (contact layer) was made of petrolatum-impregnated gauze pieces applied to the affected area. The second layer consisted of a soft gauze roll over the contact layer and then secured using cotton fabric. Topical antibiotic, mupirocin was added to contact dressing in case of exudates.

Challenges faced during dressing included: increased blistering due to over-application of emollient, and the second layer meeting eroded skin causing pain. These were prevented by avoiding overflooding of emollient and making contact dressing visible. The pain was reduced by breastfeeding before dressing. Blood investigations were avoided. Neonate remained hemodynamically stable without sepsis and under multidisciplinary care. Family members were counselled by neonatologists, psychologists, dermatologists, and geneticists.

**Figure 9:**
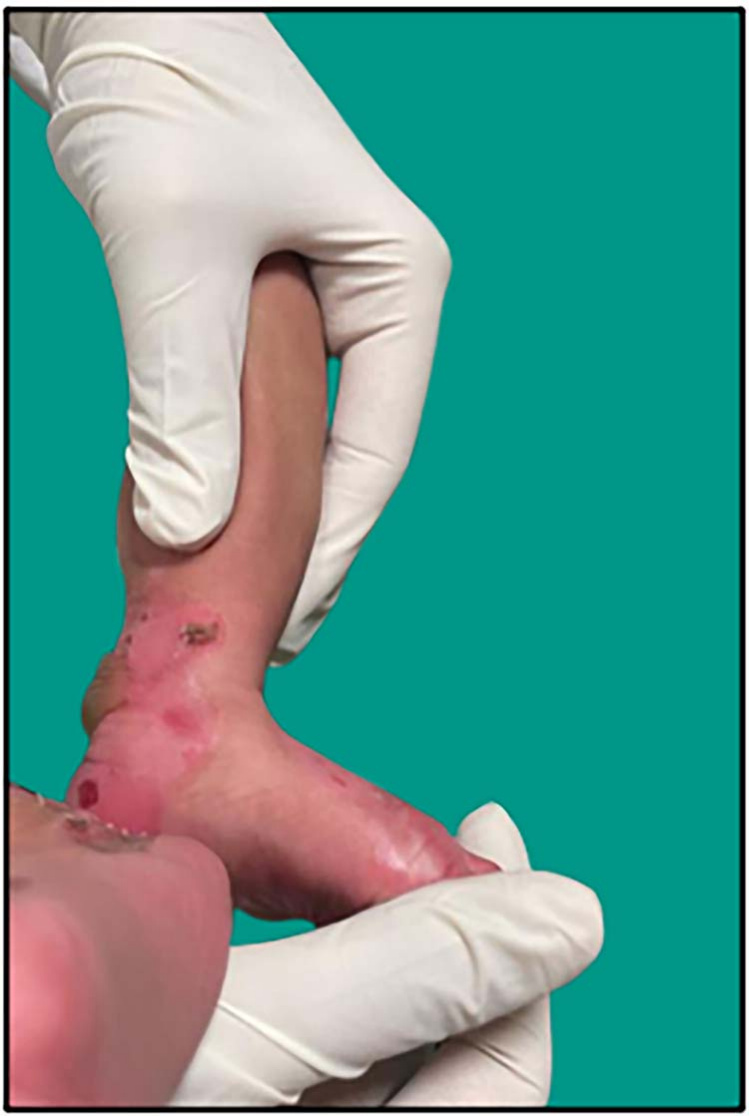
Healing of lesions of the neonate without scarring.

Parents were taught to manage the flare-ups as repeated visits to NICU incurred a lot of financial strain and absenteeism from work. At two months of age, the infant is currently 3960 g on breastfeeding with normal growth and development. The lesions have healed without scarring. (Fig. 9). The mother is under follow-up with the dermatology team.

## DISCUSSION

EB is classified into 30 subtypes based on phenotypic variation, genes involved, immunohistochemistry and mutation. It is mainly of four types depending on the layer of skin involved: EB Simplex [EBS], DEB, Junctional EB, and Kindler EB. EBS can be further subdivided into supra-basal and basal of which missense mutation in the keratin 5 and keratin 14 result in a localized or generalized variant of EBS [2].

The neonates with EBS present with signs of skin fragility, blisters, erosions and sometimes aplasia cutis congenita [3]. The diagnosis can be established by skin biopsy with IF, electronic microscopy (EM) or genetic testing. IF has certain advantages over EM in resource-limited settings including being less expensive, with provision to store samples in Michel’s medium and IF having more sensitivity (97% vs. 71%) and specificity (100% vs. 81%) [4].

There is no definitive treatment for the EB in literature. Dedicated and skilled nursing care includes preventing any trauma-induced blisters, avoidance of adhesive tape and sterile proper dressing. The blisters should be punctured with sterile hypodermic needles and the roof should not be removed. The dressing should be changed on alternate days [5]. Increased blistering at the edges of the dressing, wound maceration, worsening exudate, infection, or poor healing are often due to the overuse of emollients or improper dressing techniques. Overuse of topical antibiotics results in the selection of resistant organisms and should be avoided [6].

Specialized airbeds and temperature regulation using air conditioners at home to prevent blisters were not feasible due to financial constraints. We encouraged the use of cotton clothing, bathing with lukewarm water and the avoidance of diapers at home.

Recent studies showed not much difference in Family Dermatology Life Quality Index among different subtypes [7]. Among those diagnosed with EBS in infancy, the prognosis of EBS is good however long-term problems include abnormalities in nails, oral cavities, eyes, scarring of limbs, physical growth and depression requiring treatment [8].

This is the first reported case of a neonate with EBS born to a mother with EBP. In a questionnaire-based survey study, 58% of EB babies born to mothers with EB had blisters at birth. Blistering in babies with JEB and DEB was significantly higher than in EBS [9]. Mother was diagnosed with EBP, a rare subtype of dystrophic EB caused by mutations in collagen VII (COL7A1 gene). The possibility of additional immune-mediated factors in pathogenesis is supported by clinical improvement with cyclosporin A [10].

Inherited EB is transmitted usually as an autosomal dominant or autosomal recessive disease, depending on the EB subtype. Most EB phenotypes have only one mode of genetic transmission. In our index case, both the mother and neonate have different subtypes of EB. Though spontaneous mutations for the autosomal dominant disease are not uncommon in EBS and are also seen in a minority of cases of DEB [11], this can be postulated as a possible explanation considering no other family member being affected other than the mother-infant dyad. The affection of protein is also different in both these subtypes (Keratin in EBS and Collagen VII in DEB) which was confirmed on IF. Only genomic testing can confirm the mutation and provide a definitive explanation for this rare entity. This was not possible due to financial constraints and is planned for follow-up.

Parents’ involvement and multidisciplinary approach right from NICU admission play a crucial role in the management. In low and middle income countries, the challenges faced during treatment and the social and financial support of the families must be reported on a larger scale warranting the urgent need for cost-effective prevention and treatment modalities.
